# Study protocol: combining experimental methods, econometrics and simulation modelling to determine price elasticities for studying food taxes and subsidies (The Price ExaM Study)

**DOI:** 10.1186/s12889-016-3277-5

**Published:** 2016-07-19

**Authors:** Wilma E. Waterlander, Tony Blakely, Nhung Nghiem, Christine L. Cleghorn, Helen Eyles, Murat Genc, Nick Wilson, Yannan Jiang, Boyd Swinburn, Liana Jacobi, Jo Michie, Cliona Ni Mhurchu

**Affiliations:** National Institute for Health Innovation, School of Population Health, University of Auckland, Auckland, New Zealand; Department of Public Health, University of Otago, Wellington, New Zealand; National Institute for Health Innovation and Department of Epidemiology and Biostatistics, School of Population Health, University of Auckland, Auckland, New Zealand; Department of Economics, University of Otago, Dunedin, New Zealand; Department of Epidemiology and Biostatistics, School of Population Health, University of Auckland, Auckland, New Zealand; Department of Economics, The University of Melbourne, Melbourne, VIC Australia

**Keywords:** Food taxes, Food subsidies, Food policy, Price elasticities, Food pricing, RCT, Modelling, Bayesian, Virtual supermarket

## Abstract

**Background:**

There is a need for accurate and precise food price elasticities (PE, change in consumer demand in response to change in price) to better inform policy on health-related food taxes and subsidies.

**Methods/Design:**

The Price Experiment and Modelling (Price ExaM) study aims to: I) derive accurate and precise food PE values; II) quantify the impact of price changes on quantity and quality of discrete food group purchases and; III) model the potential health and disease impacts of a range of food taxes and subsidies. To achieve this, we will use a novel method that includes a randomised Virtual Supermarket experiment and econometric methods. Findings will be applied in simulation models to estimate population health impact (quality-adjusted life-years [QALYs]) using a multi-state life-table model. The study will consist of four sequential steps:

1. We generate 5000 price sets with random price variation for all 1412 Virtual Supermarket food and beverage products. Then we add systematic price variation for foods to simulate five taxes and subsidies: a fruit and vegetable subsidy and taxes on sugar, saturated fat, salt, and sugar-sweetened beverages.
2. Using an experimental design, 1000 adult New Zealand shoppers complete five household grocery shops in the Virtual Supermarket where they are randomly assigned to one of the 5000 price sets each time.
3. Output data (i.e., multiple observations of price configurations and purchased amounts) are used as inputs to econometric models (using Bayesian methods) to estimate accurate PE values.
4. A disease simulation model will be run with the new PE values as inputs to estimate QALYs gained and health costs saved for the five policy interventions.

**Discussion:**

The Price ExaM study has the potential to enhance public health and economic disciplines by introducing internationally novel scientific methods to estimate accurate and precise food PE values. These values will be used to model the potential health and disease impacts of various food pricing policy options. Findings will inform policy on health-related food taxes and subsidies.

**Trial registration:**

Australian New Zealand Clinical Trials Registry ACTRN12616000122459 (registered 3 February 2016).

## Background

There is a growing call to implement structural interventions that create a more supportive food environment for healthier food choices [1–3]. In particular, health-related food taxes and subsidies are attracting increasing research and policy attention [4]. Mexico and Hungary have junk food taxes and a number of countries have soft drink taxes, including France and Mexico [5, 6]. There is emerging evidence from these countries showing that these taxes are effective [7]. A recent observational study using data on beverage purchases in Mexico (*n* = 6253 households) showed an average 6 % reduction in purchases of taxed beverages compared to the previous year (-12 ml/capita/day) [8]. Experimental work also suggests likely benefits of soft drink taxes [9].

Several systematic reviews examining the effectiveness of health-related food taxes and subsidies have recently been published [10–17]. In 2014, Thow et al. conducted a review of the effectiveness of food taxes and subsidies including empirical randomized controlled trials, simulation modelling studies, surveys and laboratory studies [16]. The authors concluded that fiscal interventions can be effective in promoting healthier food choices, and soft drink taxes and healthy food subsidies seem most effective [16]. However, the authors note that there remains much uncertainty about the effects of fiscal food policies, particularly because of a lack of high quality evidence.

Randomised controlled trials (RCTs) are the gold standard to obtain evidence on the impact of health interventions, including food pricing strategies. However, trials are problematic to conduct when testing strategies that affect whole populations [12]. Consequently, most food pricing trials in the literature are conducted in controlled settings such as worksite cafeterias or vending machines [12]. Evidence from these studies does not provide much insight into the effects on total household food purchases (including non-taxed or subsidised foods), which is crucial to estimate the net impact of a health-related food tax or subsidy on health. For example, a saturated fat tax may reduce consumption of saturated fat (as it did in Denmark [18]), but also increase consumption of sugary foods. Therefore, we need trials that capture a broad range of food purchases, ideally supermarket trials (as this is where people in high-income countries buy most of their food). There are some high-quality supermarket experiments available in the literature, but these studies are limited to subsidies [19–22], and do not report fully disaggregated impacts on all food items.

Because food pricing RCTs are difficult to conduct, evidence on health-related food taxes and subsidies to date mostly relies on uncontrolled before and after studies, natural experiments, and (most notably) simulation modelling studies. Simulation modelling uses econometric estimates of price elasticities (PEs) and mathematical equations to estimate the effects of taxes and subsidies [23–27]. PEs of demand measures the percentage change in purchased quantity or demand for a product (food) with a 1 % change in price. Own-PE refers to changes in demand for a food due to changes in its own price; cross-PE refers to changes in demand for a food in response to price changes in another food [28]. For example, increasing the price of full-fat milk will decrease purchases of full-fat milk (own-PEs) and may increase purchases of low-fat milk (its substitute; cross-PE).

A recent systematic review of US studies on the PEs for sugar-sweetened beverages, fast food, fruits, and vegetables estimated own-PEs to be -1.21, -0.52, -0.49 and -0.48 respectively [15]. The authors stated that while the evidence base in this field is growing, more studies are needed to improve the precision and applicability of PEs. In particular, it is important to have studies with better groupings (e.g., separate groupings for sugar-sweetened and artificially sweetened soft drinks) and that provide individual-level data to provide evidence on differential effects across population groups [15]. Another recent systematic review aimed to combine the evidence from simulation modelling studies and investigate the association between fiscal food pricing policies and changes in food consumption, health and disease outcomes and potential differences between socio-economic groups [13]. An important finding of this review was the low to moderate quality of most included studies. For example, more than half of the identified studies did not consider cross-PEs and none validated their model [13].

In New Zealand, Ni Mhurchu et al. developed PEs for 24 commonly consumed food groups using food expenditure data from national household economic surveys, Food Price Index (FPI) data and an Almost Ideal Demand System (AIDS) approach [29]. The authors reported own-PEs ranging from -0.44 (ready-to-eat food) to -1.78 (poultry) which were generally higher than those reported in other countries [15]. The authors highlighted the need for better country-specific PEs, particularly because the available national household expenditure surveys were small (6028 households compared to 93,000 for comparable UK studies) and covered a relatively short time period (four years compared to 15 years in UK studies). Moreover, the New Zealand surveys do not record food prices and purchase quantities therefore requiring matching with FPI data to estimate the PEs which could introduce bias [29].

To provide good quality evidence for decision makers on the likely impact of food price changes, we need to measure own-PEs and cross-PEs accurately and precisely. However, current food PEs (particularly cross-PEs) are imprecise and impede estimation of likely population health impacts of fiscal policies [28]. Few studies have comprehensively approached this issue experimentally and those that have were too small to measure own- and cross-PEs with precision [9, 12, 20, 30]. We propose to overcome all the above mentioned issues by employing an experimental approach to generate data on varying food price and consumption to then feed into econometric modelling to estimate (specific and accurate) food PEs. By using an experimental design, we will be able to generate large price variations for the food groupings of most relevance to public health policy.

## Methods/Design

The Price Experiment and Modelling (Price ExaM) study aims to: I) derive accurate and precise food PE values; II) quantify the impact of price changes on quantity and quality of discrete food and beverage groups and; III) model the potential health and disease impacts of these specified food taxes and subsidies. To achieve this, we will use a novel combination of methods including a virtual experiment, econometric methods to estimate PEs, and public health intervention modelling to estimate health gain (i.e. quality-adjusted life-years (QALYs)) and health costs saved using a multi-state life-table (MSLT) model. The overall study involves four sequential steps: selection of fiscal policies and specification of price sets (Step 1); randomised Virtual Supermarket (VS) experiment (Step 2); estimation of PEs (Step 3); and estimating health impacts of food taxes and subsidies (Step 4). These four steps are outlined in detail below, constituting the remainder of this Study Design and Methods section.

### Step 1: Selection of food tax and subsidy policies and specification of price sets

To generate robust PEs, there needs to be price variation in the VS experiment (i.e. Step 2), but not so much price variation as to be unrealistic. One could just create random variation in all prices. However, to focus the price variations on the ‘foods that matter most’, we undertook a specification of the tax and subsidy policies of most relevance to modelling for public health purposes (Step 4), and generated price sets with maximal variation about the foods most likely to be included in tax and subsidy interventions. This process should improve the accuracy and precision of the own- and cross-PEs most relevant to the selected policies.

#### Selection of food tax and subsidy policies

We selected five policy options: sugar tax, saturated fat tax, salt tax, sugar-sweetened beverage (SSB) tax and a “fruit and vegetable” subsidy (Table 1). Within each policy option, we specified a low and high variant (e.g. a 20 % or 40 % tax), and within two of the five food categories (i.e. SSBs and fruit and vegetables) we further specified variations of exactly what was included (e.g., just sugary carbonated soft drinks to all sugary drinks).

**Table 1:** The five selected food pricing policy options

Type of tax / subsidy	Scenario	Tax or subsidy % or amount	Included foods/drinks
1. SSB tax	a) Sweetened sugary beverage (SSB) Tax	20 and 40 % options	Sugar-sweetened carbonated soft drinks
	b) SSB + sugar-sweetened (SS) fruit drinks, SS energy drinks, SS sports drinks tax	20 and 40 % options	Above, plus: Cordials and fruit drinks, Sports drinks, Energy drinks, Powdered drinks
	c) SSB + SS fruit drinks, fruit juices, SS energy drinks, SS sports drinks tax	20 and 40 % options	Above, plus: Fruit Juices includes apple, orange, grapefruit, grape etc.
	d) Fizzy drink tax	20 and 40 % options	Sugar-sweetened carbonated soft drinks Sugar free carbonated soft drinks Fizzy energy drinks
	e) Tax on all sugar containing beverages	20 and 40 % options	Sugar-sweetened carbonated soft drinks Electrolyte drink Energy drinks Cordial bases Fruit drinks Fruit/veg juice Flavoured water Hot drink mixes (Milo, hot chocolate, cocoa and cereal beverages etc.) Flavoured milk Powdered drinks
2. Fruit and vegetable (FV) subsidy	a) Fresh FV only	20 and 40 % options	Fresh fruit Fresh vegetables (excluding potato products)
	b) Fresh FV + frozen	20 and 40 % options	Above, plus: Frozen vegetables (plain) Frozen fruit
	c) Fresh FV + frozen + + dried + canned	20 and 40 % options	Above, plus: Canned legumes Fruit in juice/syrup Dried fruit Canned vegetables Corn (can) Legumes (can) Tomatoes (can)
3. Saturated fat tax	Starting point: doubling the price of butter	$2/100g^a^ (low) or $4/100 g (high) tax options	All processed (non-fresh) foods; excluding olive oil and avocado oil
4. Sugar tax	Starting point: doubling the price of raw sugar	$0.4/100 g (low) or $0.8/100 g (high)	All processed (non-fresh) foods containing sugar
5. Salt tax	Starting point: quadrupling the price of raw salt (results in approximately 4 to 8 % tax on bread)	$ 0.02/100 mg sodium (low) or $0.04/100 mg sodium (high) per 100 gram of product	All processed (non-fresh) foods containing salt

^a^All dollar values are in New Zealand Dollar (2015 1NZ$ = 0.697US$, http://stats.oecd.org/index.aspx?queryid=169)

#### Specification of price sets

**Fig. 1:**
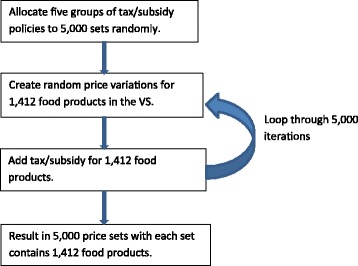
Procedure to generate 5,000 price sets for the Virtual Supermarket

In total, we generated 5000 price sets (see Fig. 1), from which each participant in the VS experiment will be randomly assigned to, without replacement, during one of their five shopping experiences. The price sets are divided into five broad categories in line with the five tax/subsidy policy options. Within each price set, the price of *all* 1412 food products in the VS will vary randomly, with some correlation within food categories and independence between categories. The amount of taxes/subsidies added on top of these price sets, in most cases, leads to larger price variations. The variations in price for particular products will depend on the particular policy option. For example, we will have more variations in SSB prices in the SSB tax scenarios. Using this approach, Price ExaM will provide reasonably precise PEs for any food group which can be used to test any food pricing policy, but will maximise precision about PEs for the most likely policy options. Further details about the procedures to generate the price sets are documented in Appendices 1 and 2.

### Step 2: virtual supermarket experiment

**Fig. 2:**
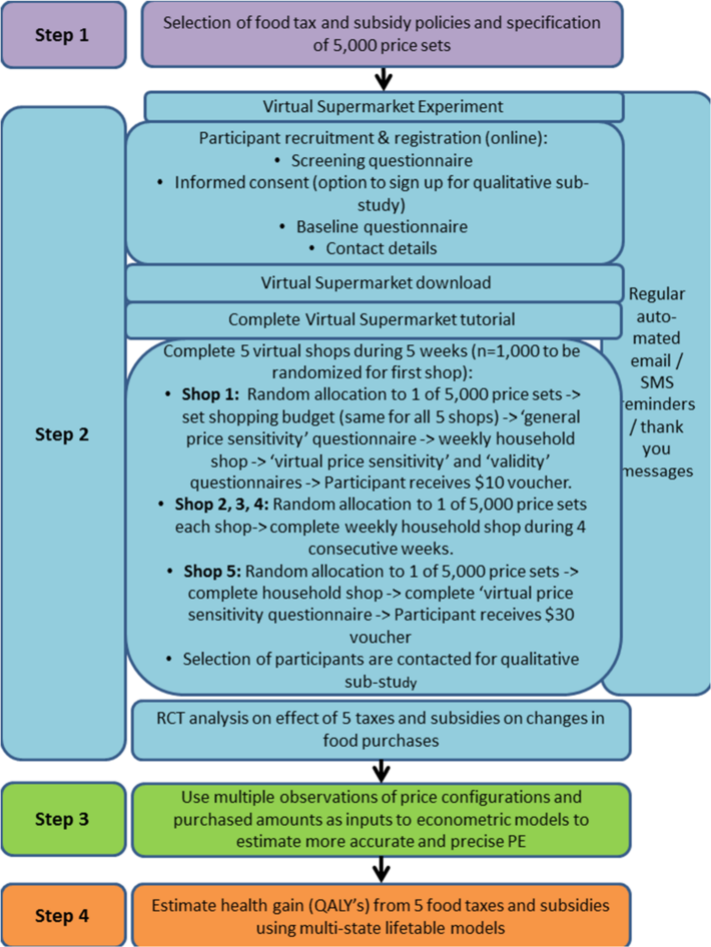
Detailed flow diagram of the price ExaM study

Step 2 uses an experimental design where study participants will be randomised to one of the 5000 price sets developed in Step 1, at each of the five shops in the VS (see below). The details of the experimental phase are outlined below and in Fig. 2.

#### The virtual supermarket

The New Zealand VS creates a realistic three-dimensional computer simulation of a real supermarket mirroring the in-store environment of one of the leading supermarket brands in New Zealand [31]. A recent validation study confirmed that people’s shopping behaviour in the VS was similar to that performed in real life [31].

**Fig. 3:**
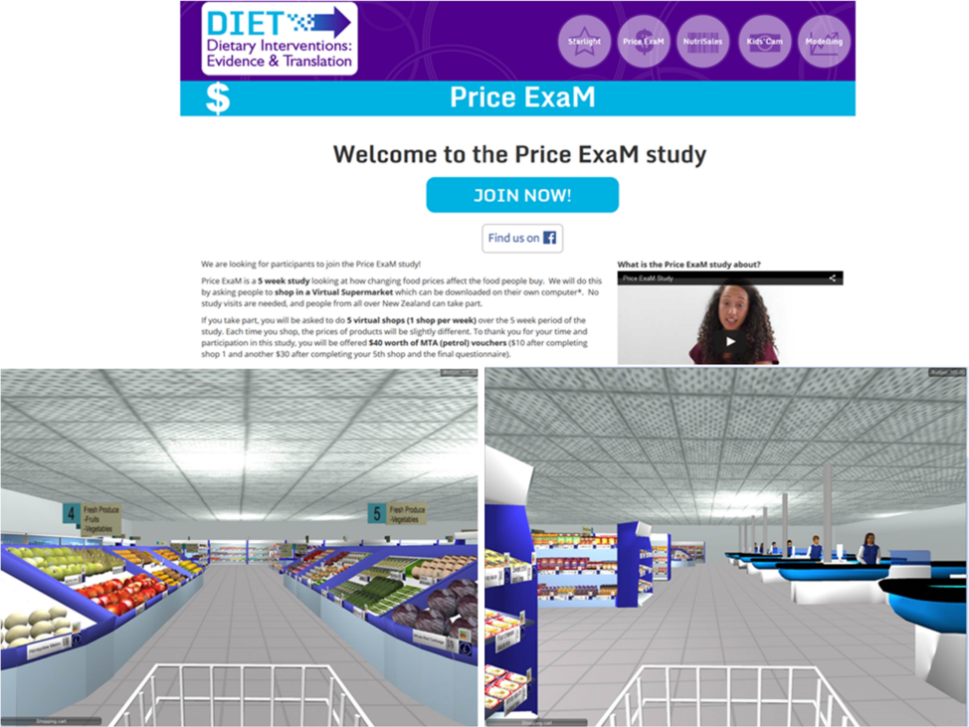
Price ExaM Participant page and images of the virtual supermarket

The front end of the VS contains 1412 unique food items positioned on supermarket shelves (Fig. 3). Photographs of real products are used to compose product images and food prices are clearly marked on the shelves and both pop up when participants hover their computer mouse over a product. Behaviour in the VS has been designed to simulate purchasing in real life; participants navigate through the supermarket with a shopping trolley using their computer cursor keys. They select groceries by clicking on them and the product then appears in their trolley. While shopping, a list of selected groceries is visible, including the price and total amount of money spent thus far. Participants can use this list to change products or delete them altogether from their trolley. A set of checkouts is located at the far end of the supermarket where participants virtually pay and leave the supermarket. The application captures all purchases made.

The VS is linked to all product information, including food prices and nutritional composition. For this study, all packaged products were linked to Nutritrack [32], a database of the brand-specific nutrition information for New Zealand packaged food products. Food composition data for fresh foods and alcohol were derived from the New Zealand Food Files generic food composition database [33]. The 5000 price sets developed during Step 2 were all stored on a server and link to the VS using an Application Program Interface (API) (see more details below).

#### Eligibility

Participants eligible for inclusion in the study are: adults (18 years of age or older) with access to a computer or laptop with an internet connection, an email address, who are confident in basic computer skills, speak and read English, contribute to household grocery shopping and will be available during the study period. Only one person per household can participate.

#### Recruitment

Participants are recruited from the general New Zealand population, using advertising in newspapers, magazines, on social media, radio, websites, flyers and posters, existing networks and word of mouth. In total, we aim to randomise 1000 participants. We will include as many Māori (indigenous New Zealanders) as possible. We are engaging with Māori networks to develop Māori-targeted recruitment videos and will continue to engage with these networks where appropriate.

#### Experimental procedures

A flow diagram of the experimental phase is provided in Fig. 2. Participants complete the entire study online, using our Price ExaM website (https://diet.auckland.ac.nz/content/priceexam-join). The website contains all forms (registration, consent, questionnaires), the VS software for download, and instruction material (including manuals and videos) (Fig. 3). Following registration and consent, participants are able to download the VS onto their computer. Next, they will login with their email address. At their first login, participants will be asked to complete a tutorial where they must find and purchase six products in the VS. This tutorial helps participants to become familiar with the software. After completing the tutorial, participants are asked to set their shopping budget. This budget is the approximate total amount they can spend during each shopping task and the amount must cover food purchases and beverages (including alcohol) only (i.e., the VS does not contain non-food products such as soap or toothpaste normally found in a supermarket). Previous studies revealed that participants find it difficult to estimate their weekly supermarket food purchasing budget, therefore we provide them with a range of examples. Participants must spend at least 50 % of their allocated budget and may overspend to a maximum of 125 % to warrant a realistic shop. On each of the five shopping occasions, participants will be instructed to buy the groceries for their household for the coming week just as they would in real life. They will be asked to complete their shops roughly one week apart from each other. We assume a five-week period is long enough to capture staple purchases that people might not buy every week. Participants will receive regular automated text and email reminders during the study to remind them about completing the virtual shops and questionnaires.

#### Randomisation

For each of the five shops, participants will be randomly allocated to one of the 5000 price sets, without replacement, using a random number generator. Therefore, participants do not receive price sets within the same policy option for each shop and randomisation is not stratified (i.e., it is completely random).

#### Blinding

Participants will be informed that this study aims to measure shopping behaviour. They will also be informed that prices in the VS vary and that they are able to see the prices when they shop. However, participants will not be told: (i) how the prices vary; (ii) that these price changes relate to fiscal policy options; (iii) and that the study relates to health outcomes.

#### Outcome measures

The main output of the VS experiment will be the multiple observations of price configurations and purchased amounts which can then be used to calculate three main outcomes:

A. **Differences in food purchases between the broad food pricing policy options** (Table 1). The following food purchasing measures will be compared between the broad food pricing options that participants have been randomised to:
  - Mean quantity (adjusted for household size) of key nutrients, including saturated fat (g/100 g and % energy), total sugar (g/100 g and % energy), sodium (mg/100 g) and energy content (total KJ and KJ/100 g) of the total shopping basket.
  - Nutrient profiling score of the total shopping basket calculated using the Food Standards Australia New Zealand (FSANZ) nutrient profiling standard [34].
  - Mean quantity (g/ml) (adjusted for household size) of food groups most impacted by the five fiscal food policies (e.g., SSB’s, snacks, fruit and vegetables, etc.).

Statistical analyses will be performed by the study statistician at the end of the experiment, using SAS version 9.4 (SAS Institute Inc., Cary, NC, USA). All randomised shops for all participants will be included in the analysis, on an intention-to-treat basis. Random effects mixed model will be used to test the differences in effect of policy options on food purchase outcomes, adjusting for important socio-demographic variables (sex, age, and ethnicity). Correlation between repeated shopping data collected from the same participant will be taken into account using a random subject effect.

B. **Food price elasticities** estimated using the VS output and traditional econometric and/or Bayesian modelling methods (see Step 3 below).
C. **Health gains/losses and net healthy system costs** for each tax/subsidy policy calculated through the BODE^3^ (Burden of Disease Epidemiology, Equity & Cost-Effectiveness Programme) MSLT “DIET” model (under development; similar to the BODE^3^ Tobacco model [35]). This model estimates QALYs and net health system cost impacts arising due to changing diets that then lead to changing disease incidence rates and then to changing mortality and morbidity rates (see Step 4 below).

For each of the three main outcomes, we will examine (pre-specified) interactions by important population groups, including by ethnicity (Māori versus non-Māori), sex and age, noting the limited power. Furthermore, we will measure price sensitivity; shoppers tend to be heterogeneous with regard to their attention and reaction to price and price promotions, which we will account for in our models. Lichtenstein et al., developed and validated a questionnaire to measure price sensitivity [36] from which we will include questions on the factors ‘value consciousness’, ‘price consciousness’ and ‘price mavenism’ (mavenism is how attuned one is to market and price conditions). Price sensitivity questions will be asked right before the first shop (both to measure price sensitivity and to prompt participants to focus on food prices in the VS) and after the first and last shop (where questions will be worded to reflect price experiences during the virtual shopping task).

#### Ethics approval

The experimental phase began in February 2016 and will continue for 10–12 months until recruitment targets are reached. Ethical approval was obtained from the University of Auckland Human Participants ethics committee on 10/11/2015 (reference 016151) for three years.

### Step 3: estimation of price elasticities

#### Overview

Following the VS experiment, in Step 3, we will use the output data (i.e. multiple observations of price configurations and purchased amounts) as inputs to econometric models to estimate more accurate and precise PEs. We base our analysis on the Bayesian estimation approach to exploit results from the previous literature. This method also provides us with a powerful tool to address censoring in the data. Estimation of the demand system via the Bayesian approach requires prior assumptions about the model parameters as an input into the estimation process. As the PEs are a function of the parameters in the demand models, this enables us to incorporate PEs from the literature (The New Zealand SPEND study [29]) as part of the prior assumptions for the model parameters using the functional relationship between PEs and model parameters and additional constraints on the range.

The prior information, summarised in terms of the prior distributions of the parameters, is combined with the information from the data, summarised in the likelihood, to obtain the posterior distributions of the model parameters as well as the PEs. Point estimates and standard deviations of the model parameters and PEs are then computed from these posterior distributions.

#### AIDS model

The empirical analysis to obtain PE estimates from the data is based on an AIDS model [37], for example of the following form:

1 $$ {\mathrm{w}}_{\mathrm{i}}={\upalpha}_{\mathrm{i}}+{\displaystyle \sum_{\mathrm{j}=1}^{\mathrm{n}}}{\upgamma}_{\mathrm{i}\mathrm{j}} \ln {\mathrm{p}}_{\mathrm{j}}+{\upbeta}_{\mathrm{i}} \ln \left(\frac{\mathrm{X}}{\mathrm{P}}\right)+{\upvarepsilon}_{\mathrm{i}} $$

where: w_i_ is budget share of good i, i = 1,..,n; p_j_ price of food j; X is total food expenditure; P is price index; α_i_, γ_ij_ and β_i_ are parameters to estimate; and ε_i_is the error term.

Price elasticities (PE) are calculated as follows [38, 39].

2 $$ {\in}_{\mathrm{i}\mathrm{j}}=\frac{\upgamma_{\mathrm{i}\mathrm{j}}-{\upbeta}_{\mathrm{i}}{w}_j}{w_{\mathrm{i}}}-{\updelta}_{\mathrm{i}\mathrm{j}} $$

where: $$ {\updelta}_{\mathrm{ij}}\left\{\ \begin{array}{c}\hfill =1,\kern0.75em \mathrm{i}=\mathrm{j}\hfill \\ {}\hfill =0,\kern0.75em \mathrm{i}\ne \mathrm{j}\hfill \end{array}\right. $$

Income elasticity is given by

3 $$ {\upeta}_{\mathrm{i}}=\frac{\upbeta_{\mathrm{i}}}{{\mathrm{w}}_{\mathrm{i}}}+1 $$

#### Bayesian methods

The estimation of the demand model requires an assumption of prior distributions of the model parameters. As is standard in the literature, normal distributions will be assumed for the model parameters, chosen partially due to their computational aspects. Prior means for the model parameters informing the PEs, {β_*i*_} and {γ_ij_}, will be specified with the help of two matrices of PE estimates from BODE^3^ [40] and SPEND [29], ∈ _ij_^BODE3^ and ∈ _ij_^SPEND^, respectively. The PE matrix from BODE^3^ consisted of 22 food categories with PE values adapted from the literature for developed countries. The PE matrix from SPEND was estimated from the Household Economic Survey data in New Zealand for 24 food categories.

Equation (2) will be used in connection with the PE matrices to construct priors for demand equation parameters: α_i_, γ_ij_ and β_i_ in Eq. (1). Since the number of parameters to calculate is larger than the number of prior PEs, we will also use a prior simulation approach in connection with additional assumptions to set reasonable prior means for all parameters.

We will then develop an algorithm that accommodates these priors and zero expenditures to estimate a demand system using an AIDS model as detailed from Eqs. (1, 2 and 3).

Steps to estimate the Bayesian AIDS model include:

1. Specify functional form for the demand model, which is an AIDS equation system.
2. Convert prior PE matrices to prior mean parameters in the demand model.
3. Use Monte Carlo simulation to draw values for the prior parameters from specified distributions.
4. Use Bayesian approach to combine the prior information for the parameters with the data from the VS experiment to obtain estimates for these parameters based on their posterior distributions.
5. Calculate PE values from the posterior distributions of parameters.

#### Price elasticity estimation

As part of the Bayesian model estimation we will compute point and interval estimates for PEs, both own-PEs and cross-PEs by income level and ethnicity. We will also calculate compensated/uncompensated PEs, and conditional/unconditional PEs for a complete food demand system. We will estimate a standard PE matrix as in the literature; PE matrices by specific fiscal policy; and a PE matrix for combined fiscal policy analyses in this project.

### Step 4: estimate health impacts from food taxes and subsidies

Finally, in Step 4, we will run disease simulation models (MSLT simulation models) with the two alternative methods of capturing dietary change following taxes and subsidies, namely:

- Directly inputting the average amount of each food product purchased per adult in the VS (Step 2) for each policy option, which then flows into changes in dietary risk factors.
- Using the PEs from Step 3, and then merging with a price change through to changes in food purchases and then the same flow through dietary risk factors.

The main output of the MSLT will be QALYs gained and net health system costs for the five selected fiscal policy options, for interventions (outlined taxes and subsidies) and business-as-usual (BAU, no tax or subsidy) scenarios for the entire New Zealand population alive in 2011, simulated out until death. This BODE^3^ DIET MSLT uses projected all-cause mortality and morbidity rates by sex and age for Māori and non-Māori in a ‘main’ lifetable. Running alongside this main lifetable are 17 diet-related disease lifetables, where proportions of the population simultaneously reside: coronary heart disease, stroke, type 2 diabetes, osteoarthritis, and multiple cancers (oesophageal, pancreatic, kidney, colorectal, endometrial, lung, thyroid, liver, stomach, head and neck, gallbladder, ovarian and breast). The proportion of the New Zealand population in each disease lifetable is a function of the disease incidence, case-fatality and remission (in cancers only).

The intervention effect is captured through changes in grams of food consumed between the BAU and intervention scenarios (either through directly inputting the average amount of each food product purchased per adult in the VS or through PEs, as outlined above). This will then be converted into a change in energy, fruit, vegetables, SSBs and sodium intake and the percentage of total energy from polyunsaturated fat. Energy intake will be used to calculate changes in body mass index. The change in these risk factors will then be combined with relative risks for the associations with the various diseases through population impact fractions (PIFs) that alter the incidence of the diet-related diseases.

Each diet-related disease has incidence, prevalence and case-fatality specified by each sex, age and ethnic group (Māori and Non Māori) in 2011. Remission rates were specified for cancers, but set to zero for coronary heart disease, stroke, type 2 diabetes and osteoarthritis as remission for these diseases is generally unlikely. Sex, ethnic and age-specific morbidity will be calculated for each disease using the years of life lived with disability (YLDs) from the New Zealand Burden of Disease [41], which in turn were estimated using disability weights from the Global Burden of Disease 2010 Study [42].

The net health system cost (NZD) will be the net of the intervention cost (i.e., the cost of a new law in New Zealand requiring the tax intervention [43] or paying for the subsidy programme) and any difference in projected future health system expenditure resulting from changes in disease incidence due to the interventions. Sex and age-specific health system costs (2011 NZD), will be calculated using individually linked data for publicly funded (and some privately funded) health events. Building on an existing framework for calculating the timing of health system costs [44], everyone in the model will be assigned a sex and age-specific annual cost of a citizen without a diet-related disease and not in the last six months of their life. Additional disease-specific excess costs will be assigned to people in the first year of diagnosis, last six months of life if dying of the given disease, and otherwise prevalent cases of each disease. Costs will be modelled over the lifetime of the cohort, including costs related to the diet-related diseases modelled and those not related to these diseases. This means that increased longevity due to dietary interventions contributes to increased health system costs for some cohort members.

The MSLT model is a Microsoft Excel based macro-simulation model using an Ersatz add-in to run the Monte Carlo simulations 2000 or more times. Each of these simulations involves a random draw from the probability density function about the parameters specified with uncertainty in the model. This results in central estimates for QALYs and costs with associated uncertainty intervals.

## Discussion

The Price ExaM study will provide high-quality evidence on the likely impact of health-related food taxes and subsidies by estimating precise and accurate own-PE and cross-PE values for use in modelling studies.

Good quality own-PEs and cross-PEs are lacking in New Zealand [29] and internationally [13, 15], mostly due to restraints in existing modelling and experimental methods which rely on (often weak) observational data to estimate PEs. Price ExaM aims to overcome these restraints by combining econometric, experimental and simulation modelling methods. To the best of our knowledge, this appears to be the first study globally to use such a combination of methods.

A key feature of Price ExaM is the use of the VS to objectively measure direct responses to price changes for a wide range of food items. Previous studies relied on household economic surveys and/or Food Price Index data which have important limitations including insufficient variation in price; difficulty matching food categories between consumption and price datasets; and statistically imprecise and unstable estimates for foods most relevant to public health research (e.g. cross-PEs between fruit/vegetables and foods high in salt). By exposing 1000 study participants to five different price sets, having different price sets for each participant, and by randomly varying the prices of more than 1400 foods, Price ExaM is likely to generate a uniquely and rich database allowing us to calculate PE values for many different food groupings. Therefore, Price ExaM will not only provide high-quality evidence on pre-selected food tax and subsidy options (e.g., sugar tax, saturated fat tax, salt tax, SSB tax and fruit and vegetable subsidy), but the output can also be used to examine any other fiscal policy impacting on food prices (for example a carbon tax on agricultural emissions), thus offering a unique resource for future research.

The Price ExaM study has the potential to enhance the public health and economic disciplines by introducing internationally novel scientific methods to estimate accurate and precise food PEs. These data will be used to model the potential health and disease impacts and health costs saved of various food pricing policy options. Findings will likely to be highly relevant to inform policy on health-related food taxes and subsidies.

## Trial status

Recruiting.

## Abbreviations

AIDS, almost ideal demand system; BAU, business-as-usual; BODE^3^, burden of disease epidemiology, equity & cost-effectiveness programme; Cross-PEs, cross-price elasticities; FPI, food price index; FV, fruit and vegetables; MSLT, multi-state life-table; Own-PEs, own-price elasticities; PEs, price elasticities; Price ExaM, price experiment and modelling study; QALY, quality-adjusted life-years; RCT, randomised controlled trial; SPEND, strategic pricing: effects on nutrition & disease study; SS, sugar-sweetened; SSB, sugar-sweetened beverages; VS, virtual supermarket

## Appendix 1: procedure to generate price sets for the virtual supermarket

The price sets used in the Virtual Supermarket are generated using the following procedures:

- **Step 1:** We allocate five groups of tax/subsidy policies, with 16 policy subsets, to 5000 price sets. The allocation of tax/subsidy policies are detailed in Appendix 2. In summary, we allocate 60 % (3000) of the price sets to only one policy. Namely, 15 % (750) of the price sets have no policies turned on; 15 % SSB tax; 15 % fruit and vegetables subsidy; 10 % (250) saturated fat tax; 10 % sugar tax; and 10 % salt tax. There are 20 % (1000) of the price sets that have two policies turned on. Finally, there are 5 % (250) of the price sets that have three or more policies turned on. We also create 100 price sets as reserves (e.g. to replace an invalid shop). These sets are randomly drawn from the above 5000 sets.
- **Step 2:** We create random price variations for 1412 food products in the VS. Let’s denote **P** as current food price (sourced from Nutritrack [33] 2011/2012 and online supermarkets, updated for 2014).
  1. Create Ratio1 for each of 21 food categories (e.g. fruit, vegetables, bakery, etc.), randomly selected from a uniform distribution 0.85 to 1.15.
  2. Create Ratio2 for each of 1412 food product, also randomly selected from a uniform distribution 0.85 to 1.15.
  3. Multiply the PxRatio1 × Ratio2 for all 1412 foods to create new prices (**Pnew**). This results in randomly varying prices for all foods, pre-tax/subsidy, ranging from 70 to 130 % of starting P. This allows some correlation within food categories, and independence between food categories.
- **Step 3**: We add tax/subsidy for each of 1412 food products by randomly drawing, without replacement, one of the 5000 policy sets created in Step 1. Depending on which tax/subsidy policy is turned on, the amount of tax/subsidy for each food products will be calculated accordingly (e.g. by multiplying Pnew for SSBs by 1.2 if randomly allocate to policy 1a (Appendix 2 below). That is, denoting **Pnew.ts** as food price after tax/subsidy:

Pnew.ts = Pnew × ∆ tax/subsidy

Note that the ‘∆ tax/subsidy’ is not sampled from a probability distribution, e.g. it is just ‘1.2’ for policy 1a; Step 2 generates random variation.

## Appendix 2

**Table 2:** Allocation of tax/subsidy policies to 5000 price sets used in the Virtual Supermarket

Tax/subsidy policies	N where this only policy (% of 5000)	N where they also have another policy turned on (% of 5000)
No tax or subsidy	750 (15.0 %)	750 (15.0 %)
1a. 20 % tax SSB (sugar-sweetened soft drinks only)	100 (2.0 %)	173 (3.5 %)
1b. 40 % tax SSB (sugar-sweetened soft drinks only)	100 (2.0 %)	180 (3.6 %)
1c. 20 % tax SSB+ (sugar-sweetened soft drinks, sugar-sweetened fruit drinks, sugar-sweetened energy drinks, sugar-sweetened sports drinks)	100 (2.0 %)	197 (3.9 %)
1d. 40 % tax SSB+ (sugar-sweetened soft drinks, sugar-sweetened fruit drinks, sugar-sweetened energy drinks, sugar-sweetened sports drinks)	90 (1.8 %)	187 (3.7 %)
1e. 20 % tax SSB++ (sugar-sweetened soft drinks, sugar-sweetened fruit drinks, fruit juices, sugar-sweetened energy drinks, sugar-sweetened sports drinks)	90 (1.8 %)	185 (3.7 %)
1 f. 40 % tax SSB++ (sugar-sweetened soft drinks, sugar-sweetened fruit drinks, fruit juices, sugar-sweetened energy drinks, sugar-sweetened sports drinks)	90 (1.8 %)	175 (3.5 %)
1 g. 20 % tax all soda	90 (1.8 %)	201 (4.0 %)
1 h. 40 % tax all soda	90 (1.8 %)	188 (3.8 %)
2a. 20 % FV subsidy fresh	375 (7.5 %)	764 (15.3 %)
2b. 20 % FV subsidy fresh frozen	375 (7.5 %)	742 (14.8 %)
3a. SAFA tax ($2/100 g)	250 (5.0 %)	516 (10.3 %)
3b. SAFA tax ($4/100 g)	250 (5.0 %)	548 (11.0 %)
4a. Sugar tax ($0.4/100 g)	250 (5.0 %)	443 (8.9 %)
4b. Sugar tax ($0.8/100 g)	250 (5.0 %)	409 (8.2 %)
5a. Salt tax ($0.02/100 g)	250 (5.0 %)	409 (8.2 %)
5b. Salt tax ($0.04/100 g)	250 (5.0 %)	433 (8.7 %)
6. Combination of 2 policies	1000 (20.0 %)	1000 (20.0 %)
7. Combination of 3 or more policies	250 (5.0 %)	250 (5.0 %)
Totals	5000 (100 %)	7750 (155 %)
